# Using populations of human and microbial genomes for organism detection in metagenomes

**DOI:** 10.1101/gr.184879.114

**Published:** 2015-07

**Authors:** Sasha K. Ames, Shea N. Gardner, Jose Manuel Marti, Tom R. Slezak, Maya B. Gokhale, Jonathan E. Allen

**Affiliations:** 1 Center for Applied Scientific Computing, Lawrence Livermore National Laboratory, Livermore, California 94550, USA;; 2 Global Security Computer Applications Division, Lawrence Livermore National Laboratory, Livermore, California 94550, USA;; 3 Instituto de Física Corpuscular, CSIC-UVEG, E-46980 Valencia, Spain

## Abstract

Identifying causative disease agents in human patients from shotgun metagenomic sequencing (SMS) presents a powerful tool to apply when other targeted diagnostics fail. Numerous technical challenges remain, however, before SMS can move beyond the role of research tool. Accurately separating the known and unknown organism content remains difficult, particularly when SMS is applied as a last resort. The true amount of human DNA that remains in a sample after screening against the human reference genome and filtering nonbiological components left from library preparation has previously been underreported. In this study, we create the most comprehensive collection of microbial and reference-free human genetic variation available in a database optimized for efficient metagenomic search by extracting sequences from GenBank and the 1000 Genomes Project. The results reveal new human sequences found in individual Human Microbiome Project (HMP) samples. Individual samples contain up to 95% human sequence, and 4% of the individual HMP samples contain 10% or more human reads. Left unidentified, human reads can complicate and slow down further analysis and lead to inaccurately labeled microbial taxa and ultimately lead to privacy concerns as more human genome data is collected.

Metagenomic analysis of human clinical samples is often confounded by the abundance of host genome present in the sample. This can be particularly challenging when trying to uncover a poorly characterized etiologic agent that may or may not be recoverable from a diagnostic next-generation sequencing run (Yozwiak et al. 2012; Wilson et al. 2014). Ideally each sequencer read should be examined and its relationship relative to all previously sequenced organisms accurately reported. Unfortunately, the most sensitive search needed to detect highly divergent organisms does not scale when naively examining an entire sequencing run that may total many gigabases (Zhao et al. 2012). A complementary approach is to use a metagenomic assembler; however, low coverage of individual microbial genomes can limit the potential for good quality assemblies in complex samples (Howe et al. 2014) or samples dominated by host background. Marker-based approaches present an important option for fast estimation of microbial content but only tag a small fraction of the input leaving potentially interesting fragments hidden in a large collection of unlabeled reads (Liu et al. 2011; Berendzen et al. 2012; Segata et al. 2012; Sunagawa et al. 2013; Minot et al. 2014; Tu et al. 2014). Recent efforts have demonstrated substantial progress in labeling all reads by applying ordered searches that attempt to reserve the most computationally expensive analysis for the fewest reads (Nakamura et al. 2009; Zhao et al. 2013; Byrd et al. 2014; Cotten et al. 2014; Takeuchi et al. 2014). A recent example is SURPI (Naccache et al. 2014), which maps reads to the human reference genome to subtract host reads prior to search of the GenBank NT database. DeconSeq is another tool that identifies human reads as contaminants by aligning reads to seven assembled human genomes (Schmieder and Edwards 2011) to detect human variants beyond the single reference genome. A limitation of these approaches, however, is the use of read mapping tools that organize their respective database search against each reference gene/genome independently. This presents a fundamental scaling problem as more organisms in the population are added to the database. For example, the reference database used to build taxonomic profiles for Human Microbiome Project (HMP) samples (Martin et al. 2012) used a microbial reference database totaling 7.3 gigabases (Gb), which represents only a small fraction of the sequenced microbial strains available. In the case of SURPI, although a 42-Gb GenBank NT database is searched, a best first match approach is taken, which requires careful downstream analysis to avoid overly specific calls that fail to recognize highly conserved elements.

Nonredundant search of a population of human genomes was recently shown to improve detection sensitivity using a new data structure to map reads to multiple human genomes simultaneously (Huang et al. 2013). This approach used assembled human genomes for reference but excluded microbial genomes. A related approach uses a large *k*-mer index as implemented in software tools Kraken (Wood and Salzberg 2014) and Livermore Metagenomics Analysis Toolkit (LMAT) (Ames et al. 2013). Kraken supports efficient search by mapping *k*-mers to each source genome and storing the lowest common ancestor. A highly conserved genetic region found in hundreds of genomes is efficiently identified with the lowest common ancestor (LCA) in a single search step. Recent improvements to LMAT present a similar approach but place a greater emphasis on storing more data in two ways. First, rather than store the LCA for each *k*-mer, the list of source genomes and the taxonomic hierarchy are stored—up to a user specified limited number of taxonomic identifiers (set to 200)—to improve discrimination at lower taxonomic ranks while placing an upper bound on overall runtime. When a *k*-mer maps to more genomes than allowed by the threshold, a pruning procedure retains only the higher rank taxonomy identifiers. Second, LMAT uses a larger microbial genome database, which totals >115 gigabases of genomic sequence—assembled draft and complete genomes for virus, bacteria, fungi, protozoa and mitochondrial genomes of eukaryotes. The original database required at least 620 gigabytes (GB) of DRAM, which limited its use to researchers with access to large memory machines. To improve accessibility, the software was recently tuned to exploit a combination of DRAM and NVRAM (e.g., flash drives) (Ames et al. 2014) and in addition reduced the database size to 458 GB for the full and 17 GB for the marker databases, thus allowing the software to run on substantially lower cost machines. These recent optimizations additionally enable the use of a larger cluster, where NVRAM is used as a supplemental memory resource. Scaling is supported in part by the use of NVRAM, which allows use of a much larger local single address space than is possible with DRAM and at a lower cost. For example, the 800 GB of NVRAM available per compute node in this study is commercially available at $5 per GB compared to $10 per GB of DRAM (assuming 1 terabyte of DRAM). A key benefit of NVRAM over traditional high performance disks is the ability to randomly access any part of flash memory similarly to traditional memory at orders of magnitude higher input output operations per second (IOPS) than is possible with traditional single high performance disks. RAID disk configurations and parallel file systems with multiple storage servers increase IOPS, but also increase cost and complexity. Additionally, parallel file systems based on storage area networks (SAN) greatly increase latency compared to node local NVRAM and are not suitable for our extended memory use case. NVRAM represents an important middle memory tier that allows the NVRAM to act as a supplemental memory resource in a way that is not possible with traditional spinning disk technology.

## Results

Taking advantage of a large NVRAM resource, this paper uses LMAT to present the first evaluation of a vastly expanded database of searchable reference-free human genetic variants for metagenomic search. More than 90 terabases of raw sequence data comprising 2626 human individuals from the 1000 Genomes Project (HGP) (The 1000 Genomes Project Consortium 2012) were evaluated using 1.05 million CPU hours to identify novel human genetic variants. Table 1 summarizes new databases that were created and compared, including a new database termed “LMAT-Grand,” which includes novel human variants and an expanded collection of synthetic sequence contaminants. The LMAT-Grand database is used to annotate the 18 terabases of HMP data (The Human Microbiome Project Consortium 2012a) and report microbial contents of the HGP data. The number of newly discovered human reads in HMP samples were compared with using the standard human reference genome alone (LMAT-Ref) and using all assembled human genetic data available through GenBank (Benson et al. 2013) (LMAT-GenBank). Although the vast majority of human genetic variants found outside of the human reference genome were recovered through the use of assembled GenBank genes, novel variants from the HGP were responsible for identifying as much as 40 times more human reads in the HMP data set depending on the body site. The increased sensitivity in detecting human reads confirms that the vast majority (84.58%) of the samples contained 1% or fewer human reads. Nevertheless, if looking for low abundance organisms or investigating the smaller number of cases containing large amounts of human sequence, misidentification of these human reads can lead to costly and inaccurate downstream reporting of a sample's microbial contents. New detailed genus, species, and strain-level taxonomic profiles, plasmid, and gene profiles for all HMP samples are made available along with the newly discovered human sequence and other novel sequences (see Data access). Recent work presented searches of small subsets of HGP data that look for a small number of specific microbes (Langdon 2014; Laurence et al. 2014). A search of all available sequenced microbes across the complete Phase 3 HGP data set is given for the first time to better quantify potential sources of microbial contamination. Finally, the expanded collection of searchable human sequence data is used to report 38 million new human reads found in the recently sequenced La Braña genome (Olalde et al. 2014). The new searchable databases and software are made freely available both for large memory and smaller memory machines (see Data access). The results provide a new resource for more detailed and accurate assessments of human metagenomic sequencing data.

**Table 1.**
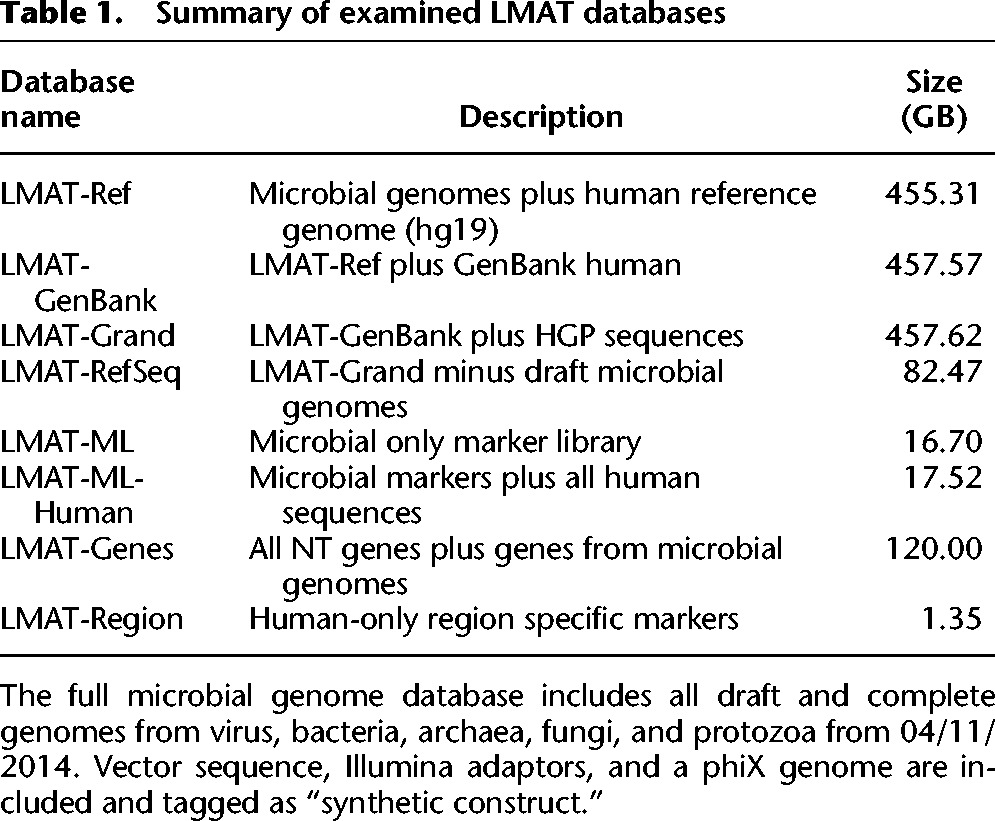
Summary of examined LMAT databases

### LMAT-Grand identifies new human sequence in HMP

The first objective was to determine the ability to detect new human sequences in metagenomic samples that were previously screened against the human reference genome using new human genomic data. In addition to the human reference genome, two sources of human genomic data were considered: human sequence from GenBank and genomic data from HGP. Adding all human genetic sequence from GenBank to the LMAT database is a nontrivial undertaking, which required downloading 72 gigabases of sequence; however, it is computationally tractable. In contrast, adding the genetic variation from 2626 human genomes was a major computational undertaking, requiring nearly half a petabyte of temporary storage and 1.05 million CPU hours on a new cluster (Catalyst) designed for data intensive computing (see Methods).

Table 2 shows a count of distinct human labeled 20-mers added from each source of human genomic DNA: the reference assembly (LMAT-Ref), GenBank (LMAT-GenBank), and HGP (LMAT-Grand). The vast majority of human 20-mers not found in the human reference assembly were found in GenBank with fewer 20-mers unique to HGP. This gives a conservative report of HGP-specific 20-mers given the strict selection criteria used to extract 20-mers from the HGP data (see Methods). Despite the smaller number of HGP-unique 20-mers and their minimal impact on database size (see column 3 of Table 1), their inclusion led to identifying substantially more human reads in select body sites in the HMP.

**Table 2.**
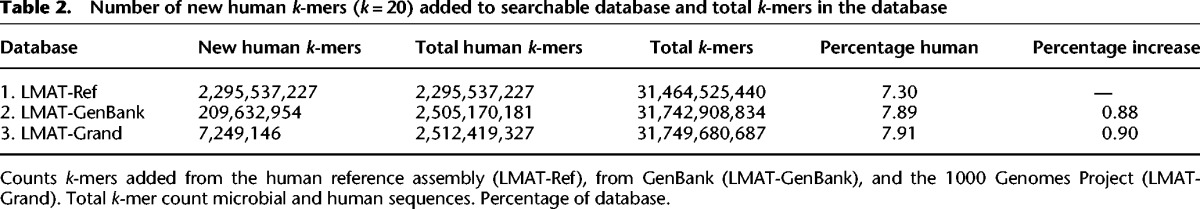
Number of new human *k*-mers (*k* = 20) added to searchable database and total *k*-mers in the database

**Figure 1.**
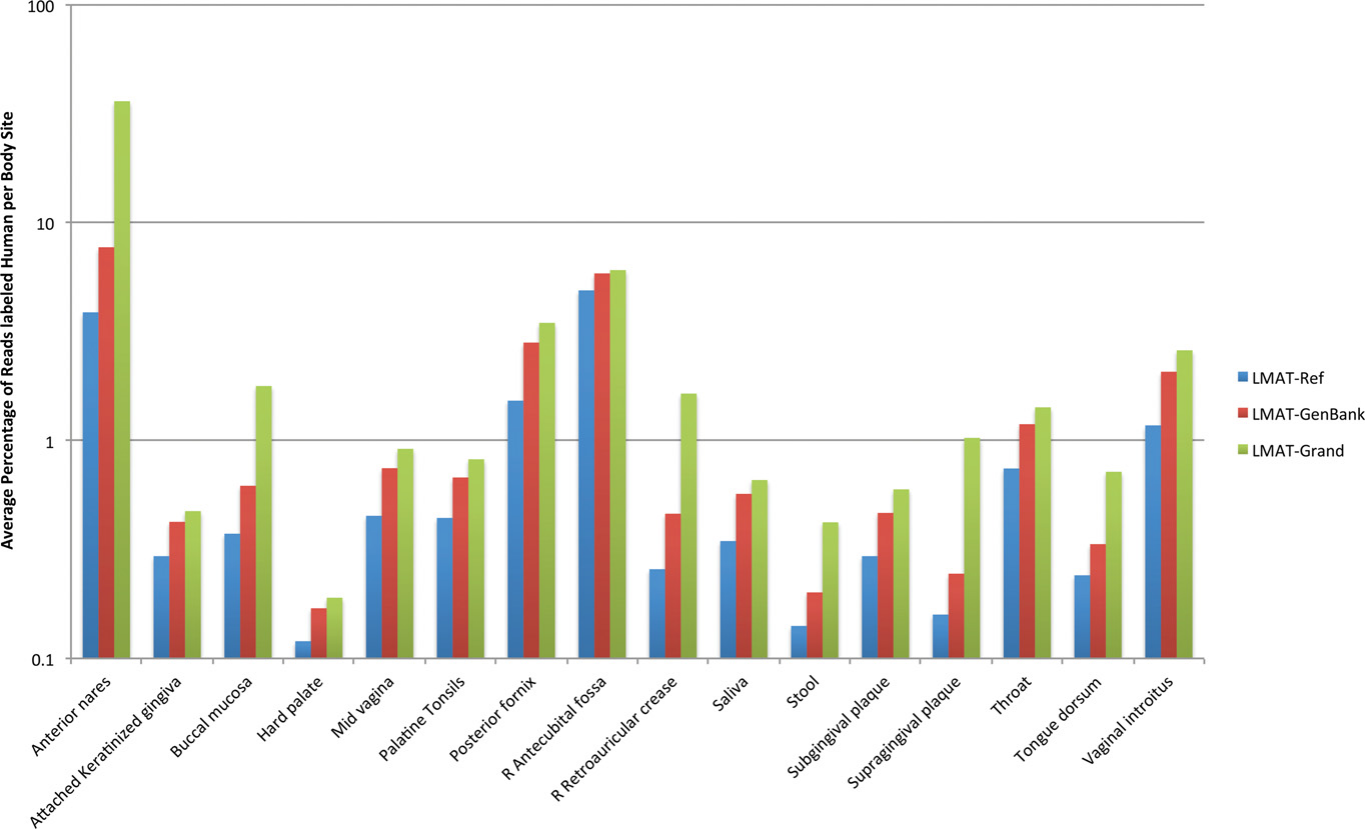
Average percentage of reads identified as human sequence in HMP samples, using LMAT-Ref, LMAT-GenBank, or LMAT-Grand by body site.

Figure 1 shows the difference in human-labeled reads across the body types sampled in the Human Microbiome Project (HMP) using 131 representative samples. All HMP samples were previously screened against the human reference genome to remove human reads using BMTagger (http://hmpdacc.org/doc/HumanSequenceRemoval_SOP.pdf). The results show that for many cases, the largest improvement is obtained through the addition of GenBank; however, in every sample, improvement in identified human reads is observed when the HGP database is included. Overall, 1.7 times more human reads were detected using HGP data over GenBank alone and 2.4 times more than from the human reference genome alone. Additionally, a collection of 1000 reads taken from world regional distinct populations were spiked into HMP mock community samples to compare detection sensitivity between LMAT-Grand and LMAT-Ref, which showed up to 1.4 times improvement in detection sensitivity (see Supplemental Material).

**Figure 2.**
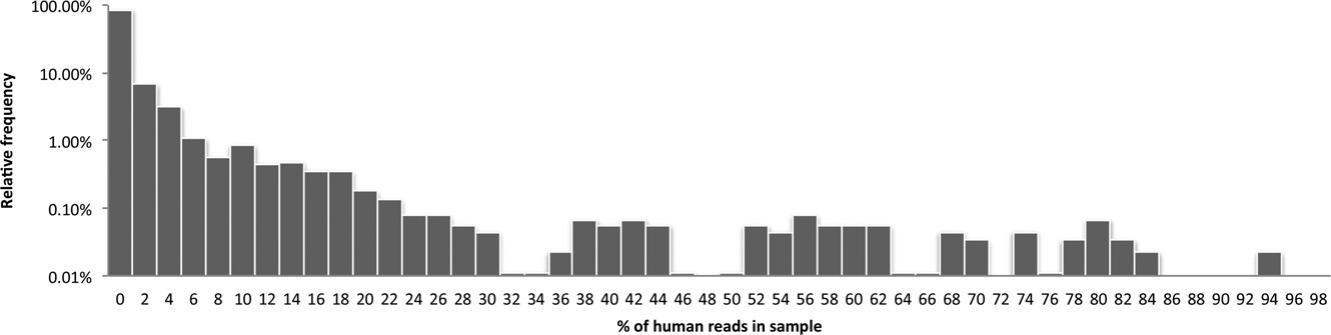
Histogram showing how often different amounts of human reads are found across the collection of sequencer runs. The *x*-axis displays human read abundance in sequencer runs in bins of 2%. The *y*-axis shows the percentage of sequencer runs with the amount of human reads specified on the *x*-axis using a log scale. The highest fraction of human reads in a sequencer run is 94% and found in one run.

Figure 2 shows a histogram for the abundance of human reads across the 9025 HMP individual sequencer runs examined using the HGP-derived database. The average amount of human DNA in each sequencer run is 2%; however, 4% of the runs contained 10% or more human reads with 75% of these samples coming from anterior nares (nostrils). Sequencer runs with an abundance of human reads (≥10%) came from different individuals and two different sequencing centers, reducing the likelihood that these newly identified human sequences come from a single contaminating source.

To confirm the newly identified human reads detected in HMP samples, BLASTN (Altschul et al. 1990) with default search settings was used to compare the reads against the NT database. Averaged over the 131 representative samples, support was found for 32% of the human reads showing eukaryote sequence (primates, other mammals, plants, insects, etc.) as the top match, including 28.5% with a top match to human. Less than 2% had their top BLAST match to bacteria, indicating a small false positive rate. These matches were examined and determined to originate from subsequences from a small number of partial bacterial genomes. Most of the reads tagged as human by LMAT-Grand had no BLAST matches using the default settings. Additional analysis that follows indicates that LMAT was more sensitive than default BLAST.

**Figure 3.**
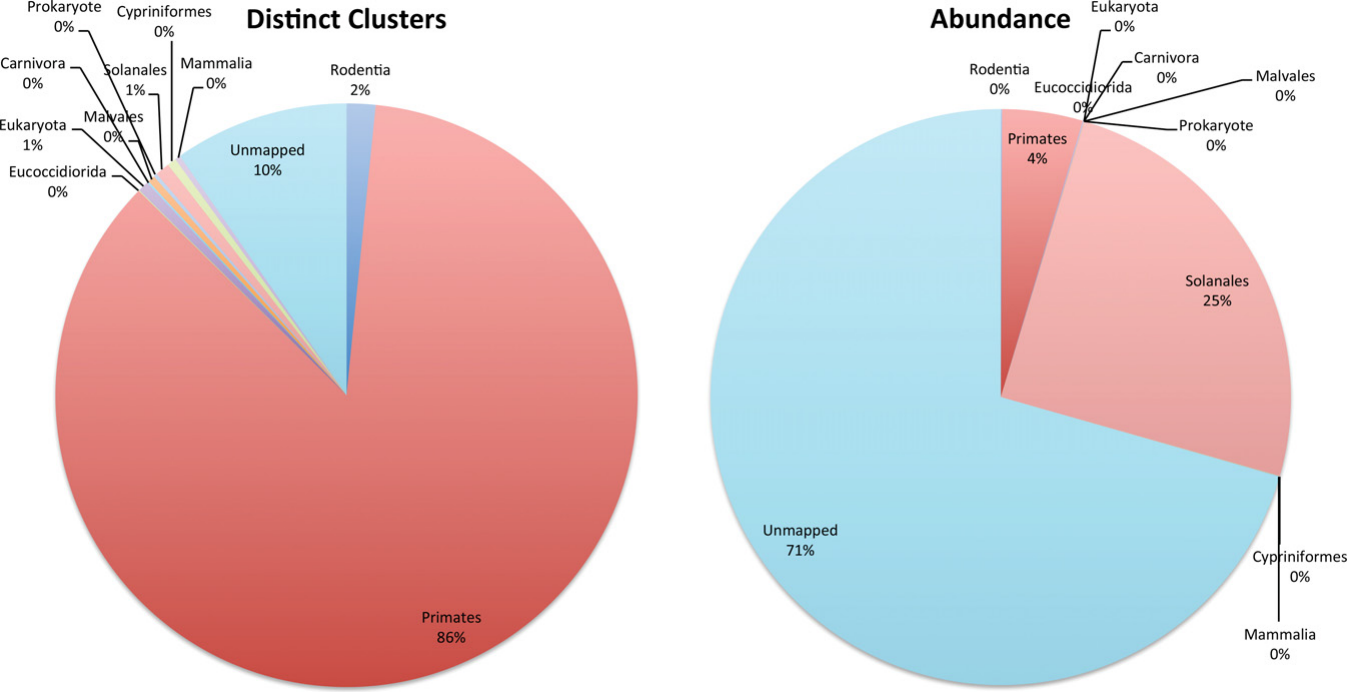
Sensitive BLAST search based assignment of reads from an HMP sample reported to have a high abundance of newly labeled human reads. The *left* panel shows the distribution of taxonomic assignments after reads were binned into clusters of similar reads. The *right* panel shows the raw abundance based on read counts for each read assignment. Taxonomic assignments with a 0% abundance label reflect percentages <1%.

A more sensitive BLAST search (using a word size of eight instead of 28 and max *E*-value of 0.01) for human reads from the sequencer run with the highest percentage of human reads was then examined (SRR059474 from an anterior nares body site). Figure 3 shows a breakdown of the reads as a percentage of the human read counts (e.g., estimated abundance) and as a percentage of read clusters after clustering reads with CD-HIT (Fu et al. 2012). As a percentage of distinct read clusters, the vast majority (86%) have a top match to a primate sequence, giving strong support for the accuracy of the newly identified human read calls. As a percentage of distinct reads, the vast majority (71%) of reads are not recognized with BLAST matches. The most abundant sequence element shows a top match to *Solanales*, primarily originating from a single highly redundant cluster. This could represent a true biological contaminant; however, the relatively high *E*-value of 0.001 indicates high sequence divergence making it difficult to confidently assert the taxonomic identity. The four most abundant read clusters (comprising 57% of the human reads) with no initial BLAST match were checked for matches in the NT database with *E*-values up to 10. One of the four clusters showed exclusive best matches to human with an *E*-value of 0.75, giving the strongest support for a human origin. In the other cases, the results were inconclusive with poor scoring matches (*E*-value ≥2.7) to bacteria and eukaryotes. The reads were checked for chimera to explain the lack of BLAST matches to human sequence, but insufficient numbers of chimera were found to explain the paucity of BLAST support. The unidentifiable human reads were also checked for their abundance across individuals in the 1000 Genomes Project collection, which show that ∼25% of the reads were associated with at least 100 individuals (see Supplemental Material).

To further determine the identity of the new human sequences, human reads with BLAST hits to RepBase (Jurka et al. 2005) were compared with comparable NT and NR BLAST hits to identify known repetitive sequences with no evidence of human sequence in GenBank but match a primate near-neighbor and show no conflicting protein matches. There were 33 distinct read clusters with a best match to a nonhuman primate sequence spanning 16 distinct repeat elements, including SINE1 elements and others (see available data). Errors in the NR database were detected in the process of comparing human reads that matched to bacterial proteins in NR. A collection of human sequences was found to be misidentified as *Leuconostoc* sp. *DORA_2* proteins (GI: 566236135, 566236136, 566236137, 566238011, 566240723). Although the BLASTX search showed 2038 human reads matched to the five different *Leuconostoc* sp. *DORA_2* protein identifiers, 2033 of the reads were found to have significant BLASTN matches to human sequences in NT. The sequences originate from premature infant guts and highlight the need to correctly recognize human reads to avoid microbial misattribution.

To further validate novel human read identification, LMAT-Grand was searched against HMP Mock Community samples as a negative control using both the staggered and even abundance Illumina samples (SRR172903 and SRR172902, respectively). Results from the staggered sample are reported as representative results. LMAT-Grand reported 4585 human labeled reads, and 669 of these could be validated with supporting BLAST alignments, indicating a small amount of human contamination even in these carefully constructed controls designed to contain only microbial DNA. However, 2135 reads were found to be likely false positives given their lower BLAST *E*-value to a microbial genome than a human genomic sequence. Although this reflects a tiny fraction of the ∼7.6 million reads, to confirm these reads were not having a disproportionate impact on over aggressive human read calling, the collection of putative false positive human reads identified in the previous set of 131 HMP samples and the mock community samples were used to remove 20-mers from LMAT-Grand that were mislabeled human (see Supplemental Material). The revised LMAT-Grand was used to re-tag all human identified sequence in the HMP samples, which reduced the number of human reads by only 1.5%. In the examined high human abundance case (SRR059474), the number of human reads was reduced by only 0.3%. Therefore, although the corrected LMAT-Grand database is made available along with the corrected collection of human reads, other output was retained.

### HMP samples contain limited world region and personal identifiable data

To consider whether newly labeled human reads could present potential privacy concerns, the reads were examined for short tandem repeat (STR) markers and world region identifiable markers. The STR detection software lobSTR (Gymrek et al. 2012) was run on the reads LMAT classified as human from the HMP data, grouping reads by subject ID across samples (combined from multiple sequencer runs) and body sites for the runs available for download, for a total of 178 subjects. An average of 25 short tandem repeats (STRs) per subject were identified, with a maximum of 54 in reads from one individual (see Supplemental Material). LobSTR reported the vast majority on Chromosome 5, one on Chromosome X, and none on Chromosome Y. Although none of these markers corresponded to 16 CODIS markers used for forensic attribution (based on positional information from http://lobstr.teamerlich.org/ystr-codis.html), it is possible that a database containing more STR markers could facilitate some level of individual identification using human reads remaining in the HMP samples after the standard procedure of read mapping to a single reference to remove human reads. The STRs were also checked against a library of newly cataloged HGP-derived STRs to determine the amount of overlap (Willems et al. 2014). There are 78 of the 178 subjects with at least 20 of the hg19 reference loci with periods of 2–6, and a few subjects have up to 33 of the reference loci. Eighty-nine of the approximately 700,000 reference loci were present in the human reads identified by LMAT from the HMP samples, predominantly on Chromosome 5, but with at least one on every chromosome except Chromosomes 18, 21, X, and Y. Although the STRs do not currently provide personal identifying information, it is conceivable that over time a sufficiently large database of human genomes labeled with personal identifying information could pose a privacy concern.

Considerable progress has been made in recent years using targeted genetic loci from the human genome to recognize shared genealogies (Elhaik et al. 2014). To investigate the potential for ethnic host identification in HMP samples, a search database was created (LMAT-Region in Table 1) to conduct world region identification from HGP genomes and ethnic codes. The LMAT-Region database is similar in structure to other LMAT databases except for two custom levels added to the taxonomy tree under the human species node. Taxonomy tree leaves are the HGP ethnic codes, and their parents are the five world regions (Africa, Europe, East Asia, South Asia, and Americas). The LMAT-Region contains human sequence only and is meant to regionally classify previously identified human reads. Supplemental Figure S3 shows ROC curves measuring sensitivity and specificity on HGP individuals, which reflect an optimistic assessment of accuracy—assuming future test individuals are drawn from the HGP population and there is relatively high coverage of the human genome present in the sample. Supplemental Figure S4 reports the ratio of total 20-mers to distinct 20-mers as a function of distinct 20-mers present in an individual human sample and provides the reference point to assess whether human region identifying reads are present in the HMP samples. The human reads for the 178 participating HMP individuals were searched against the LMAT-Region and found to yield very small numbers of region world identifiable reads (at most 76). The small number of region identifiable reads combined with the much larger number of reads used to accurately identify regions in the HGP data suggest that region identifying information from the host in HMP is not readily accessible.

### Inclusion of all draft genomes in search database improves read bin sensitivity

Including draft genomes in the reference database presents an opportunity to recover more population level genetic variation using the rapidly growing number of draft assembled genomes available through GenBank. Including draft genomic sequence must be weighed with the potential for misclassification from included contaminants in draft assemblies. This is particularly problematic for human pathogens like *Toxoplasma gondii*, where substantial amounts of unrecognized human sequence are included in the draft assemblies. The HGP data provide a valuable negative control where evidence of microbial content indicates potential false microbial identification in human samples. In response to uncovering falsely identified genomes, the “synthetic construct” taxonomy category was expanded to include contaminant sequence that would otherwise preclude the use of error-prone draft assemblies. The added sequences include an updated collection of synthetic vectors (such as cloning vectors) (Allen et al. 2008), phiX sequence (used by Illumina as a sequencing control), and Illumina adaptors. Across the representative subset of 131 HMP sequencer runs, an average of 1.7% reads were tagged as synthetic. In addition, the use of GenBank and HGP human sequence reduced the percentage of *Toxoplasma* reads from 0.37% to 0.0003%. Although the average may seem relatively low across 131 samples using only the human reference, in individual cases the percentage of *Toxoplasma* reads were as high as 11.6% and use of HGP data reduced the percentage to 0.0087%, demonstrating a powerful tool for recognizing human sequence inadvertently included in assembled microbial genomes.

An important motivation for using microbial population data is to reduce the number of unknown sequences, which require interrogation with additional computationally time-consuming methods. The hypothesis is that searching a read against all genetic variants of a strain or species will increase the number of reads that can be identified. Two metrics were used to measure the impact of adding populations of genomes—percentage of reads with no *k-*mer matches in the database (NoDbHits) and reads that have too few matches to the reference database to confidently use the assigned taxonomic label (termed LowScore). Use of the new human reference data (LMAT-Grand) did not increase the NoDbHits number but decreased LowScore reads by 2.8%, essentially allowing more human reads to be confidently labeled. The bigger impact was observed when excluding microbial draft sequence. Table 3 compares results from three databases, LMAT-Grand, LMAT-RefSeq (a subset of Grand consisting of only complete RefSeq genomes), and the previously published mapping rate for the existing read mapping–based microbial profile method available through the HMP Data Analysis Center (HMP DACC) at the web portal for accessing HMP related analysis and data (The Human Microbiome Project Consortium 2012b). The table shows a dramatic reduction in unlabeled reads from the previously reported 12% (Martin et al. 2012) down to 0.05% reads with no putative label. Thus, the number of reads with no database match is dramatically reduced by 240-fold to 139 megabases total for the HMP data set.

**Table 3.**
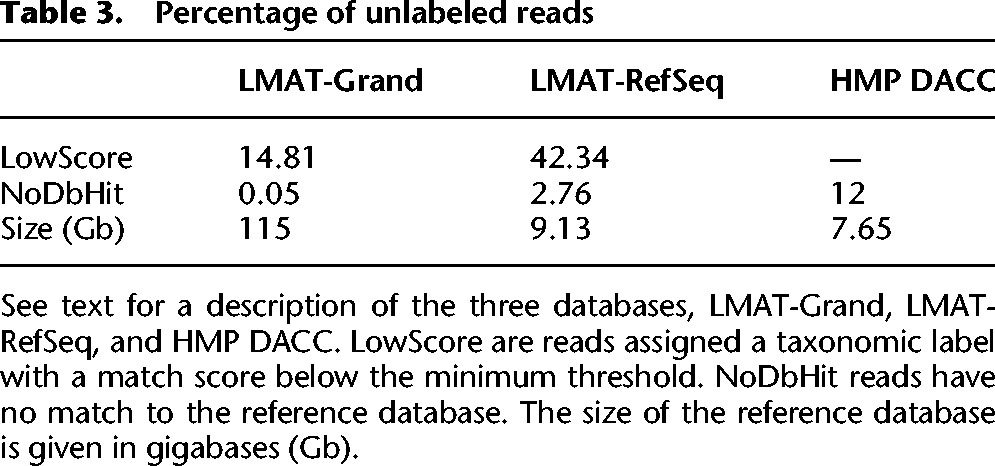
Percentage of unlabeled reads

**Figure 4.**
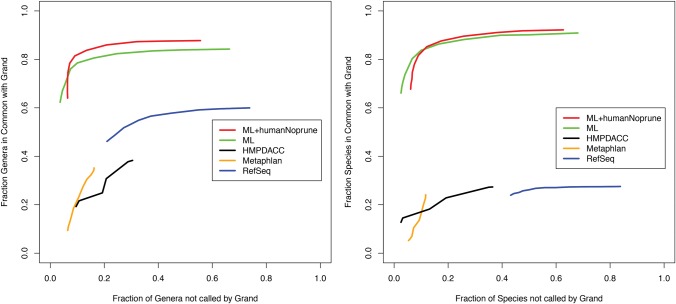
Fraction of shared genus (*left*) and species (*right*) calls. ROC curve shown using different minimum abundance thresholds to make organism calls. Different taxonomy calling methods are shown. HMP DACC, MetaPhlAn, and LMAT taxonomy calls with different database types: LMAT-RefSeq (RefSeq), LMAT-ML (ML), and LMAT-ML-Human (ML+humanNoprune).

A strikingly different microbial profile is given when relying on the RefSeq database alone as shown in Figure 4. The figure measures the amount of agreement between different databases at the species and genus level using different minimum abundance thresholds to decide organism presence. Given the larger database sizes required to store the complete collection of genome sequence, two forms of a marker library are made available to support applications on smaller memory machines. The marker library containing only microbial sequences (ML in Fig. 4) uses the LMAT-ML database described in Table 1. The marker library containing both the microbial sequences and a collection of human *k-*mers to support tagging human reads (ML+humanNoprune in Fig. 4) is represented by the LMAT-ML-Human database described in Table 1. The marker libraries are a subset of the LMAT-Grand database, retaining *k*-mers that are unique to different taxonomic ranks (Ames et al. 2013). Despite an order of magnitude smaller size (17 gigabytes versus 458 gigabytes), the marker libraries showed closer agreement to the full database compared with the RefSeq methods and the profiles available from the HMP DACC. MetaPhlAn taxonomic profiles are also available through the HMP DACC, and an updated version of MetaPhlAn profiles are included in Figure 4 as an additional profile reference point.

The species most commonly identified by HMP DACC mappings that were not detected in the same sequencer runs by LMAT-Grand were examined in detail to determine if they are HMP DACC false positive calls or LMAT false negative calls. Up to 100 reads that HMP DACC mapped to the indicated species were extracted from each HMP DACC BAM file, and BLAST searched against NT, the bacterial and viral subset of the full sequence database used by LMAT, and a database of the synthetic constructs. The fraction of BLAST hits to the indicated species was calculated for the NT and full database as well as the fraction of reads with hits to the vector database given in Table 4. BLAST matches to NT and LMAT-Grand showed very few matches to the HMP DACC species call. Up to 31% of the reads matched synthetic constructs. The HMP DACC-mapped reads to each species were run through LMAT, and the fraction of classified reads at the genus level or higher is indicated. LMAT classified up to 84% of these reads as conserved at the genus level or above. For those few remaining reads that LMAT assigned at the species level, the species with the most reads is indicated with the number of reads in parentheses. None of these species are in the reference database used by HMP DACC. The last column shows the fraction of these LMAT species-classified reads for which the LMAT species call is among the top BLAST hits. Few or none of the reads have the HMP DACC call in the BLAST matches except for candidate division TM7, which has nearly identical BLAST matches as *Saccharimonas aalborgensis,* the call made by LMAT. Therefore, the reads that LMAT does classify to species have high BLAST support for the LMAT classification. These results support the conclusion that these are false positive species classifications by the HMP DACC profiles due to a combination of overly specific calls for reads that are in fact much more widely conserved, matches to synthetic constructs that contaminate many of the draft genomes in reference sequence databases, and failure to detect the correct organism due to a lack of representation in the HMP DACC reference database.

**Table 4.**
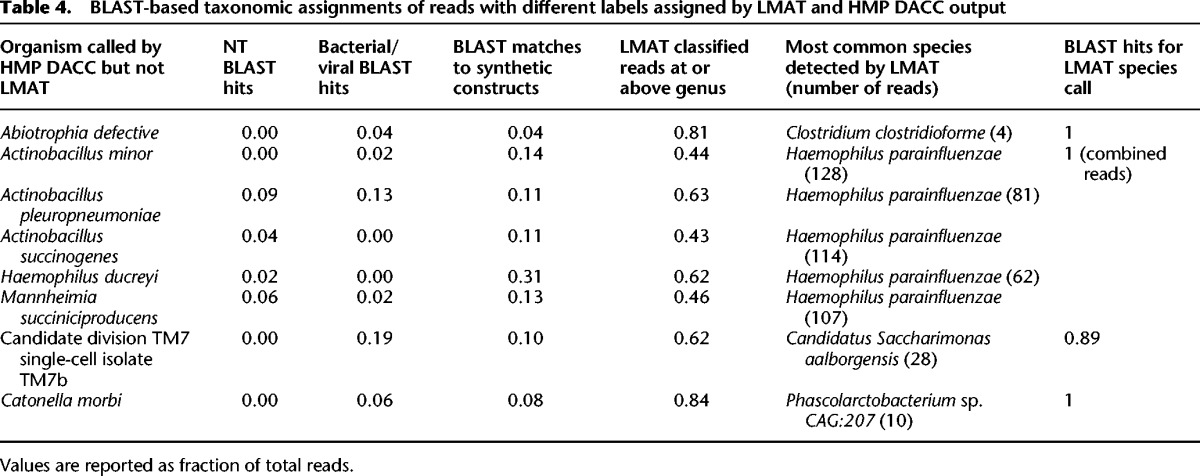
BLAST-based taxonomic assignments of reads with different labels assigned by LMAT and HMP DACC output

For the first time, profiles of the plasmid and gene content are provided for all the HMP sequencer runs. Horizontal transfer and replicate sequencing complicate interpretation of plasmid and gene profiles, particularly when the same plasmid or gene is sequenced multiple times but differently named. Since LMAT takes a “best first match” approach, the reported plasmid or gene may have equal match scores to multiple plasmids or genes. Nonetheless, taxonomic identification can be linked with gene calls to identify mobile genes of particular interest. For example, the 131 representative set was searched for plasmids known to carry the important drug resistance factor NDM-1 (*Klebsiella pneumonia* plasmid pNDM-US, *Acinetobacter baumannii* plasmid pAbNDM-1, or *Escherichia coli* plasmid p271A) (Hu et al. 2012), and then reads used to identify these plasmids were checked for their gene assignments. In total, 33 of the 131 sequencer runs indicate the presence of a NDM-1 related gene (using a minimum of 10 reads to support the NDM-1 related gene call), and in seven sequencer runs, the genes were linked with the p271A plasmid. Five of the seven were taken from oral samples (throat, palatine tonsils, and keratinized gingiva). The number of reads used to support the calls is relatively small, ranging from 10 to 94, indicating very low abundance in each sample. As a second example application, the 131 representative set was checked for human identified reads with gene assignments. Only 6% of the human reads were assigned gene calls, and 1% of these reads were associated with non-protein-coding genes, suggesting that the vast majority of detected human sequences are non-protein coding.

### Microbial content in HGP

Although HGP samples do not reflect clinical metagenomic sequencing protocols, the data provide an important “negative control” for documenting microbial content in human samples, which should have little to no microbial content with clinical relevance. Only five individuals displayed microbial content for a single organism with >10^−4^ (1.5–3.4 × 10^−3^) read abundance all for an organism identified as *Acidovorax* sp. *KKS102*, which likely reflect an environmental contaminant. Abundance is calculated using the number of reads assigned to a taxonomic label divided by the total number of raw sequencer read pairs (i.e., not quality control filtered read counts). Individual microbial contaminants could be detected in 1783 of the samples at levels below 10^−4^. Total in-sample contamination abundance was found to range between 0.1% and 1% of the reads in 1415 of the samples. The majority of these contaminants are tagged as either “synthetic construct,” reflecting known sources of contamination such as cloning vectors and adaptors, and two high rank categories “root” and “cellular organism,” reflecting reads that match to genomes found across the taxonomy tree. Although some of these reads reflect true biological conservation (such as phage represented both as a virus and integration in a bacterial chromosome) many of these reads are expected to reflect errors in the reference database. The relatively high abundance of contaminating sequences in the HGP indicates the need to accurately tag contamination so that true low frequency microbes of interest can be clearly separated and reported. Although filtering out known contaminants appears to greatly reduce the remaining number of microbial reads, clearly additional work must be done to determine whether the identified microbe is truly associated with the biological sample being examined or a persistent environmental contaminant. Although a detailed analysis of the microbial content of the HGP data is beyond the scope of this report, the data is made available for further analysis, and a brief summary of potentially interesting microbial content is included in the Supplemental Material, such as evidence of sample contamination and detection of retrovirus contaminants that were likely contributed by the immortalized cell culture used to store the HGP DNA.

### New La Bran˜a human reads

Using LMAT-Grand, the impact of detecting new human variants was examined for a completely different type of human sample—the recently published La Braña metagenome (Olalde et al. 2014) taken from a 7000-yr-old human sample, from which more than half of the reads failed to map to the human reference. With its expanded human genetic variation content, LMAT-Grand database was expected to detect substantially more human reads. All 889,666,990 unmapped reads were searched and compared (Supplemental Fig. S2, pie chart) with the Extended Data Figure 9 in Olalde et al. (2014). The previously published analysis used a random sampling of 1 million unmapped reads, which were searched against NT using BLAST, and all reads were compared against a smaller viral database to search for viruses. The new LMAT pie chart is surprisingly consistent with the predicted version previously reported. Analysis of all unmapped reads (not just a random subset) found 3% more reads could be assigned taxonomic labels than previously determined. Among the unmapped reads, 4% were newly detected to be human, although comprising a relatively small percentage of the data, constituted 37,290,232 reads. Additional analysis of the newly identified human reads will be needed to rule out the possibility of contemporary contamination. An exceptionally small percentage of the reads (0.00014%) were identified as virus and appear to consist of phage.

## Discussion

The increasing use of deep sequencing presents both a challenge and an opportunity for expanding the searchable knowledge base of circulating microbial life while characterizing microbes to support clinical applications (Fricke and Rasko 2014; Klymiuk et al. 2014). On the one hand, it is important to develop tools and databases that extract information from the new genetic variation recovered on an ongoing basis from sequencing, particularly as methods for assembly of new genomes from metagenomic sequencing advance (Albertsen et al. 2013; Nielsen et al. 2014). However, robust and high-throughput methods for error checking become critical to fully exploit the growing collection of observational data. This becomes apparent with a more exhaustive search of human genetic variants and synthetic sequences, which reveal draft microbial assemblies with contamination that can confound reference-based microbial identification. This is currently handled with LMAT by expanding the collection of tagged reference sequences. As contaminants are encountered, if they are not explicitly matched to a synthetic construct or host genome, the read is tagged as “root” or “cellular organism” to denote a taxonomically conserved element along with the list of top matches. As future work, an explicit metagenomic profile of every assembled genome is envisioned, which can be used to identify and exclude problematic assemblies. A key limitation of this approach is the need for extensive sequencing of the target environment. The current LMAT database is enriched for genomic data associated with the human target environment. Metagenomic communities isolated from other environments will continue to present additional challenges until more of the host and microbial genomes are better represented in reference databases.

The identification of human STRs in HMP samples indicate that human identification may one day be possible but only with a vastly expanded database of human genomes, which is not currently available. The absence of clear ethnic region identification markers indicate that considerable additional work would be needed to tease out any possibly important host markers in the existing HMP samples. Nonetheless, the LMAT-Region database indicates the potential to identify informative genetic host markers when there is sufficient coverage of the host genome, which could become an increasingly common feature of human metagenomic samples as depth of sequencing coverage grows.

The first published analysis of HMP data reported the use of 8.8 terabases of shotgun metagenomic sequence from 681 biological samples (The Human Microbiome Project Consortium 2012b). Since that time, many more samples have been sequenced and the current collection of 18 terabases from 1410 samples spread across 9025 sequencer runs represents a significant expansion of collected data. Comprehensive search of host and microbial populations now present scaling challenges that limit the use of traditional search tools, which rely on examining each reference genome independently. The indexing strategy used by LMAT tracks conserved sequence elements across the taxonomy tree and presents an important option for fast search, but its application has been limited by large memory requirements. Recent advances using extended memory hierarchies that use lower-cost flash drives show how exceptional performance can be achieved at dramatically lower per-compute-node costs and can support genomic search on a much larger scale than previously possible. For example, the taxonomic and gene content profiles for the 18 terabases of HMP data were completed in 37.7 h on a data intensive cluster (see Methods) and represent to our knowledge the first analysis for this large collection of HMP data.

There is a growing recognition of the challenges in using metagenomic sequencing for detecting low abundance pathogens (Naccache et al. 2013; Salter et al. 2014). The abundance of microbial content found in the HGP data further demonstrates the ongoing challenge to differentiate clinically relevant low abundance microbial content from other sources of biological contamination. Thus, the advances in methods to efficiently and accurately identify the complete profile of microbial contents within a sample must be accompanied with new strategies to avoid misattribution from unexpected sources.

## Methods

### Computational resource

The computational resource for processing the HGP and HMP data sets using LMAT is the Catalyst cluster within the Livermore Computing (LC) center. This resource provided 304 compute nodes with 24 Intel Ivy Bridge cores with 128 GB of DRAM and 800 GB PCIe attached local flash storage (NVRAM) per node. A complete copy of an LMAT database is stored on the flash drive of each node and accessed directly as if it were stored in DRAM with database contents being cached in DRAM. The open source Data Intensive Memory Map (DI-MMAP) (Van Essen et al. 2012, 2013) Linux kernel module was used to support efficient DRAM caching of NVRAM pages. Preprocessing of the HMP data for quality control did not require access to a larger memory resource and therefore, was done using a more standard Linux cluster Aztec, a 96 node (Intel Xeon 5660, with 12 cores) and 48 GB DRAM per node.

### Search database pruning

To maintain the ability to scale as more reference genome sequences are considered, the reference database uses a previously unpublished feature of LMAT that reduces the number of taxonomic identifiers pertaining to particular *k*-mers in the database. Earlier versions of the software assigned the taxonomy root to *k*-mers that have a larger number of taxonomy identifiers (IDs) than a cutoff parameter (originally set to 50). Although this approach reduces the size of the database and improves runtime, as long lists of IDs produce slow LMAT runtimes, it sacrifices accuracy by removing the pertinent taxonomic information for the *k*-mers. The new approach, referred to as “pruning,” removes all identifiers for the lowest rank first (e.g., strain) and then increasing up the taxonomy hierarchy (e.g., next species, then genus) as needed. We apply this approach at database creation, assuming that the identifiers have been preprocessed to include the identifiers for all ancestor taxons up to the LCA. Every *k*-mer that refers to more taxonomy identifiers than a specified cutoff parameter will be subjected to the pruning procedure. The taxonomy identifiers for the *k*-mer are placed in a priority queue data structure ordered by decreasing rank specificity (depth in the taxonomy tree). The data structure enables removal of all the taxonomy IDs of a particular rank until the total number of identifiers remaining in the queue is below the cutoff parameter, which becomes the reduced list of taxonomy identifiers for the *k-*mer in the database. The current database uses a cutoff value of 200 to limit the overall size of the database.

### Assembled human genome data processing

From assembled human reference genome version 38, we extracted canonical *k*-mers using Jellyfish 2.0 (Marçais and Kingsford 2011). The utility to create a searchable LMAT database merges a stream of *k*-mers in ASCII format. *k*-mers output from Jellyfish are sorted alphabetically and taken uniquely (Unix uniq utility). These *k*-mers were merged with the LMAT microbial database to form the LMAT-Ref.

To create the LMAT-GenBank database, the GenBank human identified nucleotide sequences were retrieved using the NCBI get utilities. The 72 GB of sequence was run through the same Jellyfish *k*-mer extraction procedure and merged with LMAT-Ref. The LMAT-GenBank database then was used as the starting point for creating the LMAT-Grand database as outlined below.

### Processing HGP data

To reduce the risk of errors, only “Phase 3” data as provided (ftp://ftp-trace.ncbi.nih.gov/1000genomes/ftp/analysis.sequence.index) was used for analysis, after being downloaded via ftp over the course of several weeks. Nonetheless, a major impediment to incorporating unbiased (e.g., non-reference-based) variation from raw reads is the risk of mislabeling nonhuman contaminants as novel human variants. Although extensive QC was undertaken during human genome (HG) sequencing, the potential for nonhuman contamination cannot be ruled out. Therefore, reads were initially compared against an LMAT database to identify reads with 20-mer matches to the existing LMAT human genome reference. Reads that shared no 20-mers with the existing HG reference were identified as possible nonhuman reads and were explicitly classified with LMAT using its full microbial database. Reads assigned a minimum score of one (higher confidence) for a bacterial, viral or archaea taxa were excluded. Reads assigned a score of less than one were included as human candidates with all other reads that had one or more human 20-mers present. (Reads with every 20-mer already matched to the existing HG reference were set aside and ignored.) Illumina guidelines recommend the use of minimum 30× coverage of minimum Q30 bases to infer a human SNP (http://res.illumina.com/documents/products/technotes/technote_snp_caller_sequencing.pdf). We adopted this strategy by applying a Q30 mask to all reads (e.g., all bases with *Q*-value <30 were set to N); thus, only 20-mers comprised exclusively of Q30 or greater were considered. This is much more restrictive than requiring that the single query base adhere to the *Q*-value threshold. This is an inherent limitation of operating in *k*-mer space. The computational efficiency afforded by the use of *k*-mers nonetheless expands the candidate reference set to compensate for the more restrictive quality filtering. In addition, a novel candidate 20-mer must occur at least 30 times in a human sample to be considered valid. Although this biases toward identification of repetitive sequence and reduces the variants recovered from low coverage samples, we opted to err on the side of conservative selection criteria. Finally, to further reduce possible low-level contaminants and false positives, a novel 20-mer was required to occur in at least four human samples.

We implemented a workflow to handle preprocessing and post-processing steps around LMAT using shell scripts, which would operationally integrate into a batch job scheduling environment. This workflow processed all sequence files for a particular sample as input, and produced a set of ASCII *k*-mers as output. Subsequently, we describe the steps taken within the workflow. The workflow was designed to run as a single “compute” job within the batch scheduling environment.

There were two passes of LMAT analysis on the HGP data. The first pass of LMAT ran concurrently with the preprocessing steps (via shell script pipe), where data was decompressed (unzipped) and filtered for quality using seqtk with -Q 30. In this step, LMAT ran against a precursor to the LMAT-Ref database (an earlier version of LMAT-Ref using genomic data from 9/4/2013). To support identification of potentially new human *k*-mers, we made a simple modification to LMAT to report the number of *k*-mers retrieved from the database. Any reads that had *k*-mers not matching those in the database would be considered new for the purpose of integration.

Three post-processing scripts written in awk were used to scan through output from the first pass of LMAT. In the first post-processing task, reads that contain valid *k*-mers and one or more *k*-mers that do not match the database were pooled by sample identifier and set aside to be considered as potentially novel human content. Second, reads that do not contain *k*-mers matching human in our database but show strong enough matches to other organisms were set aside for future analysis. An additional awk script counts reads that produce 100% matches to human for all valid *k*-mers.

One of the challenges facing the processing of the samples is the diversity in size. Ten samples were larger than 100 GB in total data size, so partitioning of the sample was necessary for a compute job to complete the workflow processing within a reasonable timeframe. Some human samples consist of several hundred constituent sequencer runs (input files). Our approach to partitioning each sample was to split the list of input files, assuming that the distribution of file sizes would produce reasonable partitioning for each sample. A lesson learned from this approach is that many of the individual file sizes were even too large; so a better approach (used for LMAT large runs using cluster resources) is to split the FASTA (or FASTQ) on a per-read basis. The average time per sample was 16 h, but the longest jobs took 96 h, and some remained running on the compute nodes after most jobs completed. Because our workflow specific for this process started with compressed files, partitioning raw sequence data files does not readily apply; thus, a future implementation would need to revise the workflow.

Jellyfish was used to enumerate all the canonicalized *k*-mers found in the reads determined in the post-processing to contain potentially novel human content. Following the 30× coverage requirement, for each sample, each *k*-mer must be counted at least 30 times. Distinct *k*-mers from Jellyfish are output as ASCII text, and the Unix sort utility is used to produce a set for the individual sample that can be merged with other samples.

In a process that took several hours, the 2646 input files containing sorted ASCII *k*-mers were merged (sort -m) in batches of up to 10 files per merge process. The distribution of number of *k*-mers found to occur in differing numbers of samples was examined, and a minimum of four individuals was chosen as a cutoff to balance, capturing a large number of *k*-mers while avoiding erroneous *k*-mers that may arise in a small number of samples. We used the Unix uniq tool with -c to count occurrences in the merged output and with a single-line awk script, produce such output. This set of *k*-mers generated from the 1000 Genomes Project samples are merged with the previously extracted sets of human *k*-mers and added to the searchable LMAT reference database as done in previous steps for the GenBank human gene sequence collection.

As a second pass, microbial content profiles for the HGP data were generated using the LMAT-Grand database using all candidate new human reads identified in the first pass screening of the HGP data set. The total processing time for the HGP data set was 43,600 node hours (or 1,046,400 CPU hours), which could be completed in 6 d on the Catalyst cluster.

### HMP data processing

All available HMP files were downloaded from the NCBI Sequence Read Archive (http://www.ncbi.nlm.nih.gov/bioproject/; BioProject ID 43017) on November 27, 2013, using the Aspera download utility (SRA Handbook 2014), downloaded over the course of 66 h. A total of 9,113 SRA files were converted to FASTQ using the SRA SDK 2.3.2-4 and options fastq-dump –split-files –split-spot –skip-technical –dumpbase. FASTQ-mcf revision 488 (Aronesty 2013) was used to trim reads using a list of Illumina sequence adaptors and discard reads of length less than 35. Reads were then N masked to ignore base calls with quality scores below 10, and reads with less than 35 valid bases were discarded. Paired reads were then merged to form a single contiguous read for LMAT processing. A total of 9,025 sequencer runs were available for LMAT processing after quality filtering. All steps prior to running LMAT were run on the Aztec cluster described above and took 2851 node hours, 820 of these hours were spent extracting the FASTQ format from the SRA files. A total of 10,046 node hours were used to generate the HMP taxonomic profiles, which could be completed with the Catalyst cluster in 33 h. The gene content profiles were generated in 1430 node hours and completed in 4.7 h on the Catalyst cluster.

The average runtime for LMAT-Grand, LMAT-ML, and LMAT-Gene was 17 kilobases per second per core (kbp/s), 198 kbp/s, and 63 kbp/s, respectively. This translates to ∼4.1 h to analyze a 10 gigabase data set on a 40-core machine for LMAT-Grand, 21 min for LMAT-ML, and 1.1 h for LMAT-Gene.

To compare output from different databases on a smaller subset of HMP samples, a collection of 131 sequencer runs were selected by randomly choosing up to six runs from the 19 body sites evenly divided by sex where appropriate. To examine accuracy more closely, the subset (six) of samples with existing taxonomic reports available through HMP DACC that could be matched exactly with samples in the 131 HMP set were compared (SRS052620, SRS022719, SRS053917, SRS052668, SRS057083, SRS022713). Due to the small sample size (six), a second collection of 73 samples nonoverlapping with the 131 representative set were compared and showed similar overlap; thus, the original collection was taken as a representative sample. The HMP DACC-derived profiles used a custom read mapping and taxonomy calling pipeline, and the following summary is based on the supplemental text provided by Martin et al. (2012). Briefly, a microbial database was constructed from archaeal, bacterial, viral, and lower eukaryote organisms (including draft genomes). A custom procedure was developed to remove possibly redundant genomes, based on removing genomes with 90% similarity. Reads were mapped to genomes in the database using CLC bio aligner requiring 80% identity over 75% of the read. The minimum threshold for reporting a strain present is a coverage cutoff of 0.01× depth across 1% of the strain's genome.

The most recent available version of MetaPhlAn (MetaPhlAn2 version 2.0.0 beta3; Segata et al. 2012) was used to generate MetaPhlAn profiles reported in Figure 4 using default parameter settings. For clustering, version 4.5.4 was used with default settings. NCBI BLAST version 2.2.27+ was used for all BLAST based analysis. For comparing differing taxonomic calls between different software, BLASTN was run with settings: -evalue 0.0001 -max_target_seqs 5.

### Searchable gene database

In addition to the genome databases used for organism identification, a searchable gene database was created (LMAT-Gene in Table 1) using a 20-mer index and using sequences obtained on April 22, 2014, consisting of GenBank NT genes and annotated genes from microbial genomes. Gene sequences were extracted from genomes and labeled with gene ID by combining information from the genome gff files in the NCBI genomes database (ftp://ftp.ncbi.nih.gov/genomes/), start and stop positions enumerated in the gene2accession file at NCBI (ftp://ftp.ncbi.nlm.nih.gov/gene/DATA/gene2accession.gz), and sequences in .ffn and .frn files at the NCBI genomes subdirectories for bacteria, viruses, plasmids, fungi, and protozoa using custom Perl and Unix scripts. Single gene entries in NT were extracted using the BLAST blastdbcmd with a list of gene gi's obtained from the gene2accession file. The gene database includes 23,604,714,457 nucleotides for 14,020,775 genes, and the downloadable search index is 120 gigabytes. Instead of assigning a taxonomic label to each read, an NCBI gene identifier is assigned. Genes are reported on a best first match basis and output with a minimum of 10% of the 20-mers matching a reference gene. In addition to providing gene content profiles that are independent of taxonomic assignments, the subset of reads that are assigned taxonomic identifiers with match scores above the minimum score cutoff (0) are summarized for their gene content to cross link the gene content profiles with the taxonomy content profiles.

### Plasmid identification

Evolutionary characterization of plasmids is complicated by the potential for a plasmid to be found in multiple organisms. In addition, a plasmid may be isolated under different experimental conditions, sequenced, and assigned different labels. A further complication is that draft genome sequencing projects may not identify the plasmid but include it as a segment along with other chromosomal segments. Thus, LMAT provides “rudimentary” plasmid identification, by explicitly tagging all known plasmid sequences during its database creation phase. At runtime when a taxonomic assignment is made, the list of top scoring taxonomic labels is checked for the presence of plasmids. If a plasmid is found to be among the top scoring candidates, the first encountered top scoring plasmid is chosen to replace the default taxonomic assignment. It should be noted that multiple plasmids may be assigned the same score, but only one is reported. Therefore, use of plasmids for organism identification should be considered carefully with evidence for the organism from chromosomal information. Nonetheless, it is expected that the utility to explicitly identify genetic segments that are known to be associated with plasmids will be useful for downstream analysis.

### World regional classification

The LMAT-Region database used a modified NCBI taxonomy hierarchy that designated “species,” “region,” and “ethnic” as the named ranks. Homo sapiens was retained as the sole NCBI designated species, with the five world regions included as child nodes and the 27 ethnic groups (The 1000 Genomes Project Consortium 2010) included as children nodes of their respective parent region.

Given the ethnic identification of the 2646 samples, all *k*-mers from samples with the same ethnicity that appear in four or more samples were selected as ethnic identifiers (*N* = 1–4 was considered). These *k*-mer sets were combined into a single multi-FASTA file to be input to LMAT's database creation pipeline (LMAT manual v. 1.2.4), in which each FASTA header contains only the taxonomy identifier for the ethnicity. The “pruning” threshold was set to 1 to produce *k*-mers with single taxonomy identifiers.

For validation of the database, 500 individuals were chosen from the HGP selected to be a random sampling from the 27 ethnic groups. Region classification was configured to use only reads with 100% valid *k*-mer matches to a particular ethnicity or region. Region calls were made based on taking the ratio between the first and second most abundant region calls using read counts. Regional read counts are obtained by combining the individual ethnic specific read counts. Null models were considered but did not appear to improve results and were excluded from the analysis.

## Data access

The LMAT software is freely available as open source at http://lmat.sourceforge.net, and version 1.2.4 used in this study is included as a Supplemental File. The LMAT-Grand, LMAT-ML, LMAT-ML-Human, LMAT-Genes, and LMAT-Region databases are available for download via anonymous ftp and are retrieved using the get_db.sh script included in the LMAT software distribution. The databases are listed with their actual file names, which are used as input to download a specific database: LMAT-Grand (lmat-4-14.20mer.db), LMAT-ML (kML.v4-14.20.g10.db), LMAT-ML-Human (kML+Human.v4-14.20.g10.db), LMAT-Genes (lmat.genes.7-14.db), and LMAT-Region (lmat-world-region.db). Genus, species/strain, and plasmid taxonomy profiles are available for the complete collection of HMP, and HGP sequencer runs are available as Supplemental Files. Gene content profiles for all HMP sequencer runs with a cross reference to taxonomy calls are available at ftp://gdo-bioinformatics.ucllnl.org/pub/lmat/hgp_hmp_2014/hmp.gene_cl_profiles.tar.gz. All reads with no match to the database in the HMP collection, human repeat sequences, and STRs are available as Supplemental Files. New human reads found in the La Braña genome are available through the NCBI Sequence Read Archive (SRA; http://www.ncbi.nlm.nih.gov/sra) under accession number SRX992275, and the La Braña metagenomic taxonomy profiles are provided as a Supplemental File. The HMP DACC staff is in the process of removing the human reads from the public HMP data; the reads will be made available through NCBI dbGaP (http://www.ncbi.nlm.nih.gov/gap) under study accession phs000228.v3.p1.
